# The Relationship between D’Amico and ISUP Risk Classifications and ^68^Ga-PSMA PET/CT SUVmax Values in Newly Diagnosed Prostate Cancers

**DOI:** 10.3390/curroncol31090391

**Published:** 2024-09-08

**Authors:** Ozge Ulas Babacan, Zekiye Hasbek, Kerim Seker

**Affiliations:** 1 Department of Nuclear Medicine, School of Medicine, Tokat Gaziosmanpasa University, 60250 Tokat, Turkey; 2 Department of Nuclear Medicine, School of Medicine, Sivas Cumhuriyet University, 58140 Sivas, Turkey; zhasbek@cumhuriyet.edu (Z.H.); kerimseker@cumhuriyet.edu.tr (K.S.)

**Keywords:** prostate cancer, ^68^Ga-PSMA PET/CT, D’Amico risk classification

## Abstract

Introduction: This study aimed to evaluate the relationship between pathological and clinical risk classifications in newly diagnosed prostate cancer patients, and ^68^Ga-PSMA PET/CT data and serum Prostate Specific Antigen (PSA) values. Method: A total of 203 patients who were diagnosed with prostate cancer between 2019 and 2023, who had not yet received treatment and who underwent ^68^Ga-PSMA PET/CT for staging purposes were included in this study. Results: There was a substantial correlation between D’Amico risk classification, Gleason score, ISUP classification, and the presence or absence of metastasis (*p* < 0.0001). The median SUVmax value of the prostate gland and the D’Amico risk classification were statistically significantly correlated. (*p* < 0.0001). There was a statistically significant correlation between the ISUP classification and the PSA value and prostate gland SUVmax value (*p* < 0.0001). There was a significant correlation between the median SUVmax values of the prostate gland at the time of diagnosis and the patients with and without metastases (*p* < 0.0001). According to the data obtained from ROC analysis, patients with prostate gland SUVmax values of 8.75 and above were found to have a high probability of metastasis with a sensitivity of 78.9% and a specificity of 59.05%. Conclusion: Our study showed that ^68^Ga-PSMA PET/CT is a highly effective method for staging newly diagnosed high-risk prostate cancer. The probability of metastasis was found to be dramatically increased in Gleason 8 and above. According to D’Amico risk classification, metastasis was detected in at least half of high-risk patients. Since the sensitivity of metastasis was 78.9% in patients with prostate gland SUVmax value above 8.75, we think that these patients should be carefully reported in terms of metastasis.

## 1. Introduction

Prostate cancer is the second most common type of cancer in men worldwide [1]. Similar to other cancer types, prostate cancer staging plays a critical role in determining the course of treatment and facilitating prognosis monitoring at the time of diagnosis. The majority of patients diagnosed with prostate cancer are diagnosed with localized disease and treated with radical prostatectomy or radiotherapy. 

Consequently, determining the presence of lymph node and/or distant metastases is crucial for accurate staging.

Although conventional imaging modalities for the initial staging of prostate cancer include Computed Tomography/Magnetic Resonance (CT/MR) and whole body bone scintigraphy [2], Gallium 68 (^68^Ga) Prostate Specific Membrane Antigen (PSMA) Position Emission Tomography/Computed Tomography (PET/CT), a method with higher sensitivity and specificity in distant lymph node metastasis and bone metastasis compared to conventional imaging modalities, has been used since 2012 [3,4].

PSMA is a membrane protein expressed on the surface of prostate cells. While it is expressed very little in normal prostate cells, its expression is increased 100–1000 times in prostate cancer cells [5]. Therefore, PSMA has been the target of nuclear medicine imaging in prostate cancer.

D’Amico and colleagues developed the D’Amico risk classification to predict biochemical failure after radical prostatectomy and external beam radiotherapy [6]. In this classification system, patients’ PSA levels and Gleason scores were used. Patients with PSA ≤ 10 ng/mL and Gleason score ≤ 6 were classified as low-risk patients. Patients with PSA 10–20 ng/mL and Gleason score 7 were classified as intermediate-risk patients. Patients with PSA ≥ 20 ng/mL and Gleason score 8–10 were classified as high-risk patients. Risk classification is a valuable method for determining the most appropriate treatment method for patients and for their follow-up.

The ISUP risk classification system is also a risk classification system developed for patients to receive the most appropriate treatment and follow-up, like D’Amico. According to the ISUP risk classification system, ISUP 1 is classified as Gleason ≤ 6, ISUP 2 as Gleason 3+4, ISUP 3 as Gleason 4+3, ISUP 4 as 4+4 and ISUP 5 as Gleason 9–10.

Our aim in this study was to evaluate the relationship between pathological and clinical risk classifications in newly diagnosed prostate cancer patients and ^68^Ga-PSMA PET/CT data and serum Prostate Specific Antigen (PSA) values.

## 2. Materials and Methods

A total of 203 patients who were diagnosed with prostate cancer between 2019 and 2023, who had not yet received treatment and who underwent ^68^Ga-PSMA PET/CT for staging purposes were included in this study.

The study excluded patients who were treated for other malignancies or who received treatment after being diagnosed.

Prostate biopsy results and total PSA values of the patients included in the study were recorded between the date of biopsy and the last 1 month.

In patients with Gleason score 3+3 prostate cancer with metastasis, biopsy was not repeated. However, suspicious metastases were confirmed with additional radiological imaging.

In the Department of Nuclear Medicine, ^68^Ga-PSMA PET/CT images were evaluated both visually and with software calculated standardized uptake value maximum (SUVmax) values; in addition, the presence/absence of metastases detected on ^68^Ga-PSMA PET/CT and metastasis localizations were recorded.

Prostate biopsy protocol:

All cases had prophylactic antibiotherapy. Starting from one day before the procedure, twice daily doses of 500 mg ciprofloxacin was administered orally for three days. Before the biopsy procedure, rectum cleaning was carried out through routine enema procedure. After prostate examination in left lateral decubitus position, intrarectal 5% lidocaine pomade application and periprostatic nerve block using 5 cc 2% lidocaine solution guided with ultrasound were applied to all patients. Then, 12-core biopsy specimens were obtained from the base of the right and left prostate lobes, lateral, and far remote lateral to the midline, medial and lateral parts of the apex. All these procedures were carried out using 18 Gauge 30 cm biopsy needle and automatic biopsy gun (Angiotech Tru-Core I, Gaineville, FL, USA) guided by a Diagnostic Ultrasound System 3535 (B&K Medical, Herlev, Denmark) with 7.5 MHz rectal probe.

### 2.1. ^68^Ga-PSMA PET/CT Imaging Protocol

During ^68^Ga-PSMA PET/CT examinations, the patients were administered an average of 5 mCi ^68^Ga-PSMA. All the patients stayed in the relaxation room for 45–60 min after the injection.

A General Electric Discovery PET/CT 600 device was used for imaging.

CT imaging was performed with a spiral 16-slice scanner at 120 kV and 172 mAs for attenuation correction and anatomical correlation. Three-dimensional PET imaging was performed, covering the body parts from the skull to the proximal thigh. PET imaging was conducted for approximately 2 min in each bed position. Axial, coronal and sagittal fusion images were created using the iterative reconstruction method. The maximum standardized uptake values (SUVmax) were calculated based on the PET images. An adaptive threshold setting of 42% of maximum regional metabolic activity was used for the PET images, and the region of interest (ROI) was placed within the primary tumour in the stomach by avoiding the peripheral area.

The following formula was used to calculate SUVmax:

[Activity in ROI (mCi/mL) × Body Weight (grams)] ÷ Injected Dose (mCi)

### 2.2. Statistical Analysis

SPSS version 24 software was used for statistical analysis. The median value was used to express descriptive quantitative data, while percentages were used to express qualitative data. Fisher’s exact test and chi-square test were used to compare variables. Analytical techniques (Kolmogorov–Smirnov/Shapiro–Wilk tests) and visual methods (histograms and probability graphs) were used to assess whether the variables showed a normal distribution. Descriptive analyses were performed using the median and interquartile range for non-normally distributed variables. When analysing data that was not normally distributed, the Mann–Whitney U test was employed.

The SUVmax of prostate gland values and PSA value in predicting the presence of metastasis were analysed using ROC (Receiver Operating Characteristic) curve analysis.

When a significant cut-off value was observed, the sensitivity, positive and negative predictive values were presented. A *p*-value of 0.05 was considered to indicate a statistically significant result.

## 3. Results

A total of 203 patients had a median age of 69 years (range: 31–85); 76 of 203 patients (37.4%) were metastatic and 129 (62.5%) were nonmetastatic.

**Figure 1:**
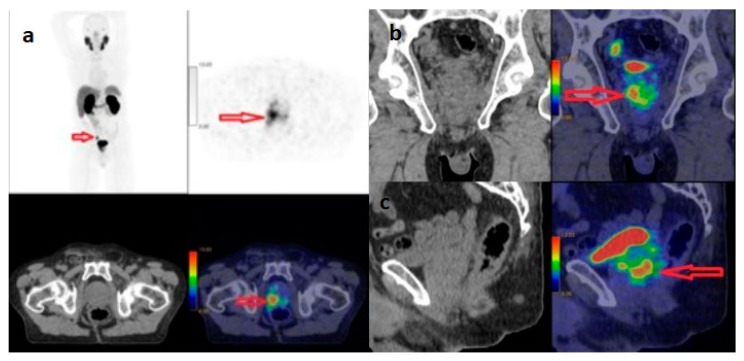
^68^Ga-PSMA PET/CT with maximum-intensity projection (**a**), and axial PET and fused axial PET/CT (**b**), coronal PET/CT (**c**) and sagittal PET/CT images of 79-year-old male with newly diagnosed prostate cancer classified as high-risk group, ISUP Grade 4, Gleason Score: 4+4, PSA: 72 µg/L. The SUV_max_ of the primary tumour was 123. Arrows indicate increased PSMA expression in the prostate gland.

According to D’Amico risk classification, 16 (7.9%) of 203 patients were low risk, 39 (19.2%) were intermediate risk and 148 (72.9%) were high risk. Of the 76 metastatic patients, 1 (1.3%) was low risk, 3 (7.7%) were intermediate risk and 72 (48.6%) were high risk. Of 127 patients without metastasis, 15 (93.8%) were low risk, 36 (92.3%) were intermediate risk and 76 (51.4%) were high risk. There was a significant correlation between D’Amico risk classification and the presence or absence of metastasis (*p* < 0.0001) (Table 1). While 48.6% of high-risk patients had metastasis, 51.4% did not. Metastasis was present in 7.7% of medium-risk patients, while it was absent in 92.3%. Metastasis was present in 6.3% of low-risk patients, but not in 93.8%. There was a statistically significant relationship between D’Amico risk classification and prostate gland median SUVmax value (*p* < 0.0001) (Table 2). In the 79-year-old patient in the high-risk group, the prostate gland SUVmax value was calculated as 123 (Figure 1). The median SUVmax value of the prostate gland of high-risk patients was 13.55, the median SUVmax value of the prostate gland of moderate-risk patients was 5.9 and the median SUVmax value of the prostate gland of low-risk patients was 5.2.

**Figure 2:**
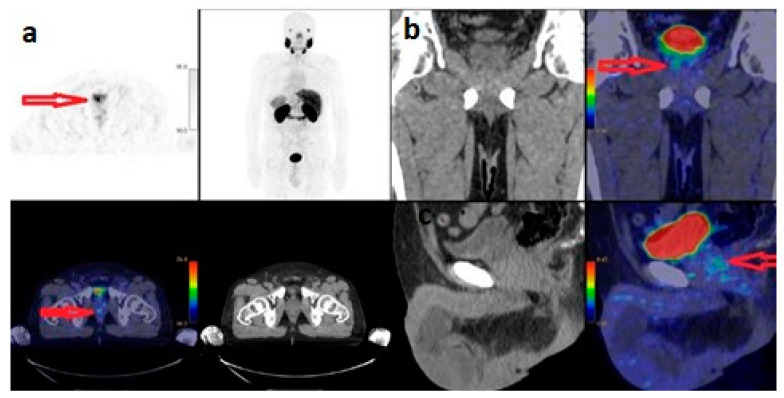
^68^Ga-PSMA PET/CT with maximum-intensity projection (**a**), and axial PET and fused axial PET/CT (**b**), coronal PET/CT (**c**) and sagittal PET/CT images of 63-year-old male with newly diagnosed prostate cancer classified as low-risk group, ISUP Grade 1, Gleason Score: 3+3, PSA: 3.59 µg/L. The SUV_max_ of the primary tumour was 3.8. Arrows indicate increased PSMA expression in the prostate gland.

**Figure 3:**
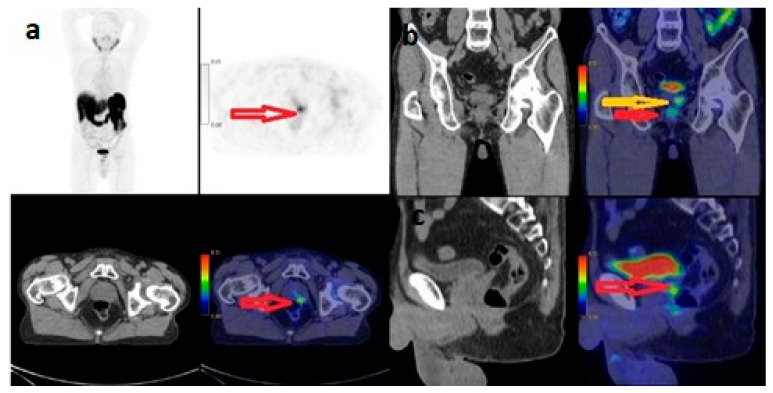
^68^Ga-PSMA PET/CT with maximum-intensity projection (**a**), and axial PET and fused axial PET/CT (**b**), coronal PET/CT (**c**) and sagittal PET/CT images of 65 year-old male with newly diagnosed prostate cancer classified as medium-risk group, ISUP Grade 2, Gleason Score: 4+3, PSA: 13.55 µg/L. The SUV_max_ of the primary tumour was 6.3. Arrows indicate increased PSMA expression in the prostate gland.

According to the ISUP classification, 51 (25.1%) of 203 patients were Grade 1, 25 (12.3%) Grade 2, 33 (16.3%) Grade 3, 47 (23.2%) Grade 4 and 47 (23.2%) Grade 5. According to the ISUP classification of 76 patients with metastases, 7 (9.2%) were Grade 1, 4 (5.3%) were Grade 2, 14 (18.4%) were Grade 3, 21 (27.6%) were Grade 4 and 30 (39.5%) were Grade 5. According to the ISUP classification of 127 patients without metastasis, 44 (34.6%) were Grade 1, 21 (16.5%) were Grade 2, 19 (15%) were Grade 3, 26 (20.5%) were Grade 4 and 17 (13.4%) were Grade 5. There was a significant correlation between ISUP classification and the presence or absence of metastasis (*p* = 0.0001) (Table 1). There was a statistically significant correlation between the ISUP classification and the PSA value and prostate gland SUVmax value (*p* = 0.0001). In a 63-year-old patient with ISUP 1 and PSA: 3.59 µg/L, the prostate gland SUVmax value was calculated as 3.8 (Figure 2). In a 65-year-old patient with ISUP 2 and PSA: 13.55 µg/L, the prostate gland SUVmax value was calculated as 6.3 (Figure 3). While 63.8% of ISUP 5 patients had metastasis, 36.2% did not. While 44.7% of ISUP 4 patients had metastasis, 55.3% did not. While 42.4% of ISUP 3 patients had metastasis, 57.6% did not.

While 16% of ISUP 2 patients had metastasis, 84% did not. While 13.7% of ISUP 1 patients had metastasis, 86.3% did not.

According to Gleason classification, 51 (25.1%) of 203 patients had Gleason 6, 57 (28.1%) Gleason 7, 48 (23.6%) Gleason 8, 30 (14.8%) Gleason 9 and 17 (8.4%) Gleason 10. Of the patients with metastases, 7 (9.2%) had Gleason 6, 17 (22.4%) Gleason 7, 22 (28.9%) Gleason 8, 18 (23.7%) Gleason 9 and 12 (15.8%) Gleason 10. Among the patients without metastases, 44 (34.6%) had Gleason 6, 40 (31.5%) Gleason 7, 26 (20.3%) Gleason 8, 12 (9.4%) Gleason 9 and 5 (3.9%) Gleason 10. There was a significant correlation between Gleason score and metastasis (*p* = 0.0001) (Table 1). While 70.6% of patients with Gleason score 10 had metastasis, 29.4% did not. In patients with a Gleason score of 9, 60% had metastasis, while 40% did not. In patients with a Gleason score of 8, 45.8% had metastases, while 54.2% did not. Of patients with a Gleason score of 7, 29.8% had metastasis, while 70.2% did not. In patients with a Gleason score of 6, 14% had metastasis and 86% did not.

The median PSA value of all patients was 22.3 µg/L (range: 1–5000 µg/L). The mean PSA value of only metastatic patients was 63 µg/L (range: 2.15–5000 µg/L) and the mean PSA value of nonmetastatic patients was 18.06 µg/L (range: 1–495 µg/L). There was a significant correlation between PSA values at the time of diagnosis and patients with and without metastasis (*p* = 0.0001) (Table 1).

The prostate gland SUVmax median value of all patients was 10.7 (1–123). The prostate gland SUVmax median value of patients with metastases was 16.5 (3.9–55.8). The median SUVmax of the prostate gland in patients without metastases was 7.2 (1–123). There was a significant correlation between the median SUVmax values of the prostate gland at the time of diagnosis and the patients with and without metastases (*p* = 0.0001) (Table 1).

**Figure 4:**
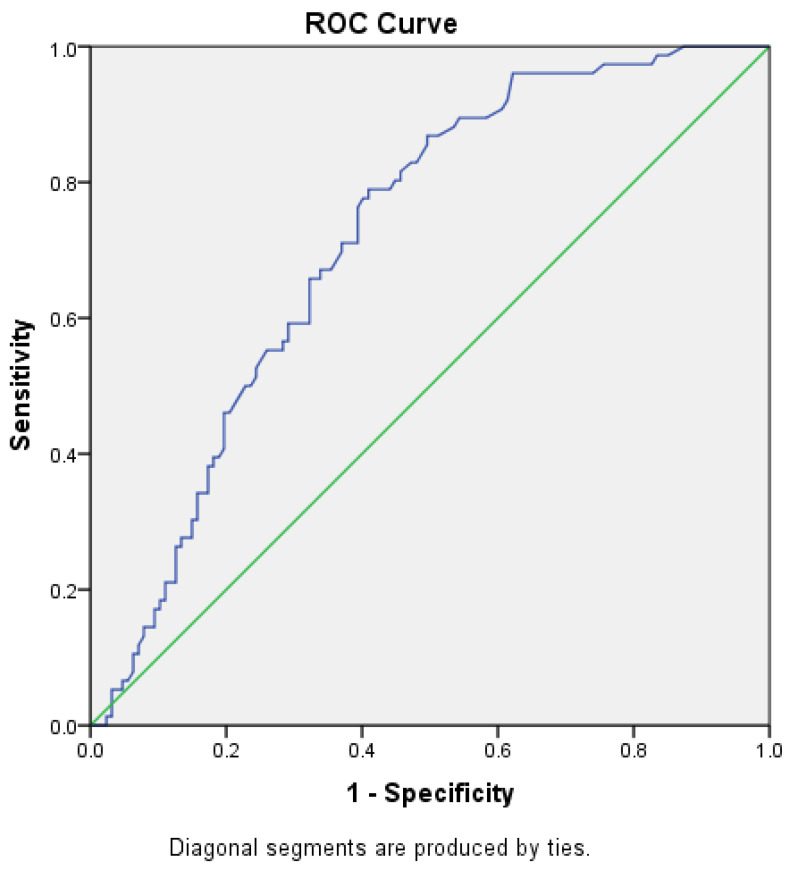
ROC analysis of prostate gland SUVmax value in showing the presence of metastasis.

According to the data obtained from ROC analysis, patients with prostate gland SUVmax values of 8.75 and above were found to have a high probability of metastasis with a sensitivity of 78.9% and a specificity of 59.05% (Figure 4) (Table 3). In patients with SUVmax values of 8.75 and above, 53.6% had metastasis, while 46.4% had no metastasis. In patients with SUVmax value below 8.75, 17.6% had metastasis, while 82.4% had no metastasis (Table 4). The positive predictive value was 53.5% and the negative predictive value was 82.4% (Table 3).

The median SUVmax value of solid organ metastasis was 4.6 (range: 1.2–54.3). The median SUVmax value of bone metastasis was 11.8 (range: 1.8–319). The median SUVmax value of abdominopelvic lymph node metastasis was 11.4 (range: 1.8–78.7).

**Figure 5:**
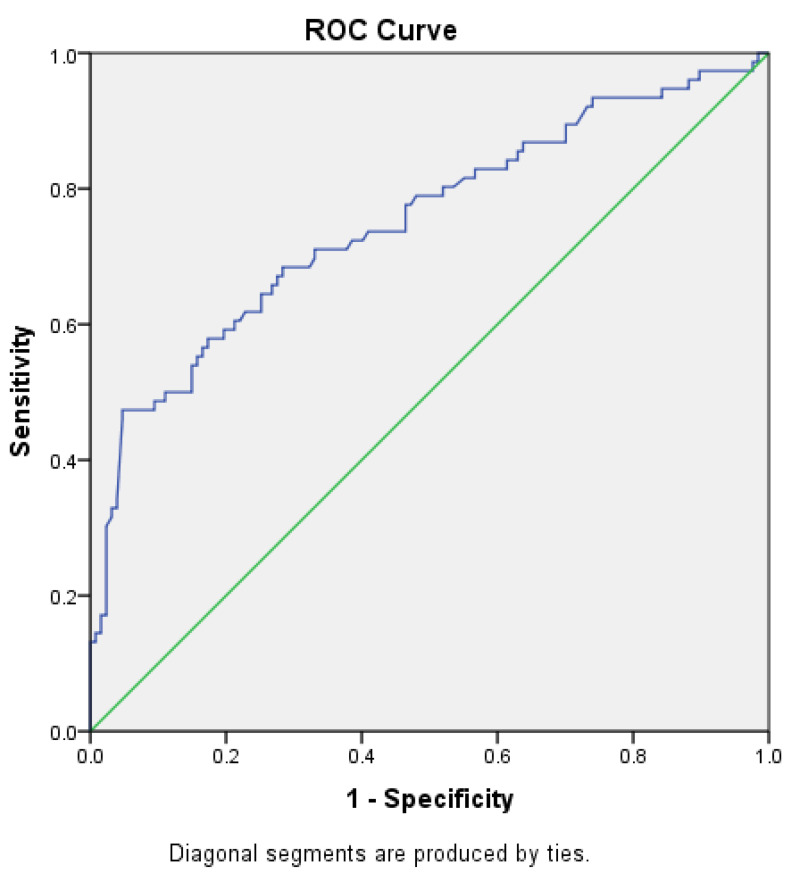
ROC analysis of PSA value in showing the presence of metastasis.

According to the data obtained from ROC analysis, it was found that patients with a PSA value of 23.55 and above had a high probability of metastasis with a sensitivity of 71.05% and a specificity of 69.9% (Figure 5) (Table 3). In patients with a PSA value of 23.55 and above, 56.3% had metastasis, while 43.8% did not. In patients with a PSA value below 23.55, only 22% had metastasis and 78% had no metastasis (Table 4). The positive predictive value was 56.25% and the negative predictive value was 79.43% (Table 3).

## 4. Discussion

For prostate cancer to be treated appropriately, the staging and the identification of metastatic lesions are crucial. Since ^68^Ga-PSMA PET/CT detects metastatic lesions in prostate cancer most sensitively, there has been intense interest in PSMA-targeted imaging.

The European Association of Urology (EAU) 2024 guideline does not recommend PSMA PET/CT in low-risk disease but emphasizes that PET/CT can be added to cross-sectional abdominopelvic imaging and bone scanning in intermediate-risk patients, but PSMA PET/CT should be added to cross-sectional abdominopelvic imaging and bone scanning in high-risk disease, if possible.

Ga^68^ PSMA PET/CT is so sensitive because PSMA is a membrane protein expressed on the surface of prostate cells and, while it is expressed very little in normal prostate cells, its expression is increased 100–1000 fold in prostate cancer cells. The fact that PSMA uptake into the cells of prostate cancer and its metastases is considerably higher than in normal cells clearly shows why ^68^Ga-PSMA PET/CT is used in staging. Immunohistochemical studies have shown that PSMA uptake is increased in metastatic, dedifferentiated and hormone-refractory tumours. PSMA expression levels in these patients are shown by the SUVmax value in ^68^Ga-PSMA PET/CT. Moreover, the level of PSMA expression is also used to determine the prognosis of the disease [7]. Increased PSMA expression can also be used to determine pre-diagnostic biopsy localization. In the study by Pepe et al., they found the SUVmax cut-off value to be 8 for the detection of prostate cancer as a result of a biopsy performed on 160 men who underwent ^68^Ga-PSMA PET/CT [8].

In our study, which consisted of a high number of newly diagnosed prostate cancers, we wanted to look at the relationship between D’Amico risk classification, which is the most effective classification system used by clinicians in staging and treatment decision making, and ^68^Ga-PSMA PET/CT data. We also wanted to look at the relationship between Gleason score and PSA levels and ^68^Ga-PSMA PET/CT.

According to the D’Amico classification, we discovered metastases in at least half of the high-risk patients in our study, but only in 6% and 7% of the intermediate- and low-risk patients (respectively). We would like to emphasize that, in compliance with EAU guidelines, PSMA PET/CT imaging should be performed on high-risk patients.

Furthermore, since we found a dramatically increased likelihood of metastasis in more than half of patients with Gleason 8 and above, we would like to emphasize that metastasis should not be predicted based on Gleason score and the patient should be scanned with PSMA PET/CT.

Ekmekçioğlu et al. found a significant correlation between the prostate gland SUVmax value and D’Amico classification. They also found a positive correlation between SUVmax values and the PSA value and Gleason score [9]. Liu et al. also found a positive correlation between the prostate gland SUVmax value, and PSA, Gleason score and D’Amico high-risk classification [10].

Prostate gland SUVmax values according to Gleason score and D’Amico classification were positively correlated in the study of Koerber et al. conducted at Heidelberg University, Department of Nuclear Medicine. Among the 104 patients in that study, the mean SUVmax value of the prostate gland was 5.97 in those classified as having low D’Amico risk, 6.98 in those classified as intermediate risk and 16.67 in those classified as having high risk [11].

Likewise, our investigation revealed a positive association between the SUVmax value of the prostate gland, and the Gleason score, PSA levels and D’Amico risk classification. In our study, we found a prostate gland SUVmax value of 5.2 in patients with low D’Amico risk classification, 5.9 in patients with intermediate risk and 13.55 in patients with high risk. These results were very similar to those of Koerber et al.

In the study by Uprimny et al. consisting of 82 patients, the median SUVmax value was found to be 8.25 in medium-risk patients and 20.5 in high-risk patients according to the D’Amico risk classification. We think that this difference may be due to the small number of patients [12].

In a study of 141 patients, Demirci et al. also found a median SUVmax value of 9.1 in high-risk patients [13].

In a study of 50 patients, Erdoğan et al. found the mean SUVmax value of the prostate gland to be 6.91 in the medium-risk patient group and 11.44 in high-risk patients [14]. Although very similar to our results, they did not find a significant correlation between SUVmax in metastatic and nonmetastatic patients in their study. However, in our study, we found a significant correlation between the SUVmax values of metastatic and nonmetastatic patients. In our study, the mean SUVmax value of metastatic patients was 16.50, while the mean SUVmax value of nonmetastatic patients was 7.2. We think that this difference is due to the much higher number of patients in our study.

In our study, 53.6% of patients with a prostate gland SUVmax value of 8.75 and above had the possibility of metastasis. Of patients with a prostate gland SUVmax value below 8.75, 46.4% had metastasis. Based on these results, we would like to emphasize that the reporting of patients with a prostate gland SUVmax value above 8.75 should be very careful in terms of metastasis. We believe that the results of our study will contribute to the literature due to the high number of patients.

In the study by Kligenberg et al., there was a moderately significant correlation between the prostate gland SUVmax values and ISUP grade [15]. In our study, there was a highly significant correlation between the SUVmax values and ISUP grade.

Demirci et al. emphasized that there was a positive correlation between the Gleason score and SUVmax values [13]. Barakat et al. found a positive correlation between PSA values and SUVmax [16].

Basha et al., in a study of 173 patients, found a median SUVmax value of 16.9 in metastatic patients and a median SUVmax value of 5.4 in patients without metastasis [17]. They also found a positive and significant correlation between the PSA value and metastasis. According to our research, the median SUVmax value for patients with metastatic disease was 16.5, while the median value for patients without metastatic disease was 7.2. PSA values and metastasis showed a strong positive correlation in our investigation. We believe that, because the number of patients in the Basha et al. study was relatively similar to ours, this is the reason why the results of their study were so similar to ours.

In contrast to our research, the Harsini Harding et al. study, which included 25 patients, did not discover any association between the PSA value and Gleason scores, and the existence or absence of metastasis [18]. We believe this to be a result of the study’s small patient population.

## 5. Limitations of the Study

The biggest limitation of our study was that it was retrospective and we could only access the information in the patients’ files at the time of diagnosis. In addition, we could not perform a prognosis study because we could not follow up with the patients.

## 6. Conclusions

Our study showed that ^68^Ga-PSMA PET/CT is a highly effective method for staging newly diagnosed high-risk prostate cancer.

According to D’Amico risk classification, metastasis was detected in at least half of the high-risk patients. According to D’Amico risk classification, ^68^Ga-PSMA PET/CT should be used for staging in high-risk groups.

According to ISUP Grade 3 and above, metastases ranging from 42% to 63% were detected. The probability of metastasis was found to be dramatically increased in Gleason 8 and above. It was also found that the incidence of metastasis increased as the PSA value increased.

Since the sensitivity of metastasis was 78.9% in patients with prostate gland SUVmax values above 8.75, we think that these patients should be carefully reported on in terms of metastasis.

## Figures and Tables

**Table 1:** Association of metastasis according to Gleason, ISUP, median PSA, median SUVmax and D’Amico risk classification.

		Metastatic			Nonmetastatic			*p* Value
		N (%)	SUVmax (Median)	PSA (Median, Min–Max)	N (%)	SUVmax (Median)	PSA (Median, Min–Max)	
D’Amico Classification	*Low Risk*	1 (% 6.3)	6.8 (6.8–6.8)	5.65 (5.65–5.65)	15 (% 93.8)	5 (1–8.7)	6.72 (3.59–12.4)	*p* = 0001 *
	*Medium Risk*	3 (% 7.7)	20 (6.3–34.8)	12.95 (11.7–13.92)	36 (% 92.3)	5 (1–34.4)	12.75 (1–19)	
	*High Risk*	72 (%48.6)	16.5 (3.9–55.8)	83.9 (2.15–5000)	76 (% 51.4)	11.5 (1–123)	25.8 (3–495)	
ISUP Scoring	*ISUP 1*	7 (% 13.7)	8.1 (6.1–28.5)	24 (5.65–307.4)	44 (% 86.3)	5.7 (1–41)	13.35 (3.59–91.7)	*p* = 0001 *
	*ISUP 2*	4 (% 16)	27.4 (9.4–35.5)	17.6 (12.95–201)	21 (% 84)	5.3 (1–31.4)	13 (1.38–81)	
	*ISUP 3*	14 (% 42.4)	20.2 (6.2–40.5)	100 (28.1–5000)	19 (% 57.6)	14.7 (3–45.7)	29.7 (1–495)	
	*ISUP 4*	21 (% 44.7)	16.7 (4.2–52.5)	100 (3.24–1816)	26 (% 55.3)	11.5 (1–123)	20.4 (6–82.6)	
	*ISUP 5*	30 (% 63.8)	16.25 (3.9–55.8)	43.06 (2.15–1848)	17 (% 36.2)	17.1 (1–68.5)	32.6 (3–314)	
Gleason Scoring	*Gleason 6*	7 (% 13.7)	8.1 (6.1–28.5)	24 (5.65–307.4)	44 (% 86.3)	5.7 (1–41)	13.35 (3.59–91.7)	*p* = 0001 *
	*Gleason 7*	17 (% 29.8)	20 (6.2–38.9)	100 (12.5–5000)	40 (% 70.2)	8.15 (1–45.7)	17.45 (1–495)	
	*Gleason 8*	22 (% 45.8)	16.9 (4.2–52.5)	100 (3.24–1816)	26 (%54.2)	11.5 (1–123)	20.4 (16–82.6)	
	*Gleason 9*	18 (% 60)	15.55 (3.9–31.5)	34.46 (2.15–1455	12 (%40)	13.95 (1–39.1)	23.9 (3–285)	
	*Gleason 10*	12 (% 70.6)	26.9 (4.9–55.8)	63 (6.16–1848)	5 (% 29.4)	26.4 (8.3–68.5)	36 (18.06–314)	

*: *p* < 0.05, significant.

**Table 2:** Median PSA values and median SUVmax values in D’Amico risk classification.

D’Amico Classification	Prostate Gland SUVmax (Median) (Min–Max)	PSA Değeri (Median) (Min–Max)
Low Risk	5.2 (1–8.7)	6.48 (3.59–12.4)
Medium Risk	5.9 (1–34.8)	12.8 (1-19)
High Risk	13.55 (1–125)	36.2 (2.15–5000)

**Table 3:** Sensitivity, specificity, PPV and NPV of prostate gland SUVmax and PSA values for detecting metastasis.

	Sensitivity	Specificity	Positive Predictive Value (PPV)	Negative Predictive Value (NPV)
SUVmax of prostate gland ≥8.75	% 78.9	% 59.05	% 53.5	% 82.4
PSA values ≥23.55	% 71.05	% 69.9	% 56.25	% 79.43

**Table 4:** PSA and SUVmax cut-off values in detecting metastasis.

PSA Values	Metastatic (n)	Nonmetastatic (n)	Total
<23.55	22 (%20.6)	85 (%79.4)	107 (%100)
≥23.55	54 (%56.3)	42 (%43.8)	96 (%100)
**SUVmax Values**			
<8.75	16 (% 17.6)	75 (% 82.4)	91(%100)
≥8.75	60 (% 53.6)	52 (% 46.4)	112(%100)

## Data Availability

The data presented in this study are available on request from the corresponding author.
